# Longitudinal Recordings Reveal Transient Increase of Alpha/Low-Beta Power in the Subthalamic Nucleus Associated With the Onset of Parkinsonian Rest Tremor

**DOI:** 10.3389/fneur.2019.00145

**Published:** 2019-03-07

**Authors:** Jan Hirschmann, Omid Abbasi, Lena Storzer, Markus Butz, Christian J. Hartmann, Lars Wojtecki, Alfons Schnitzler

**Affiliations:** ^1^Medical Faculty, Institute of Clinical Neuroscience and Medical Psychology, Heinrich Heine University, Düsseldorf, Germany; ^2^Medical Faculty, Center for Movement Disorders and Neuromodulation, Heinrich Heine University, Düsseldorf, Germany

**Keywords:** tremor, subthalamic nucleus, neuronal oscillations, local field potentials, Parkinson's disease

## Abstract

Functional magnetic resonance imaging studies suggest that different subcortico-cortical circuits control different aspects of Parkinsonian rest tremor. The basal ganglia were proposed to drive tremor onset, and the cerebellum was suggested to be responsible for tremor maintenance (“dimmer-switch” hypothesis). Although several electrophysiological correlates of tremor have been described, it is currently unclear whether any of these is specific to tremor onset or maintenance. In this study, we present data from a single patient measured repeatedly within 2 years after implantation of a deep brain stimulation (DBS) system capable of recording brain activity from the target. Local field potentials (LFPs) from the subthalamic nucleus and the scalp electroencephalogram were recorded 1 week, 3 months, 6 months, 1 year, and 2 years after surgery. Importantly, the patient suffered from severe rest tremor of the lower limbs, which could be interrupted voluntarily by repositioning the feet. This provided the unique opportunity to record many tremor onsets in succession. We found that tremor onset and tremor maintenance were characterized by distinct modulations of subthalamic oscillations. Alpha/low-beta power increased transiently immediately after tremor onset. In contrast, beta power was continuously suppressed during tremor maintenance. Tremor maintenance was additionally associated with subthalamic and cortical power increases around individual tremor frequency. To our knowledge, this is the first evidence of distinct subthalamic LFP modulations in tremor onset and tremor maintenance. Our observations suggest the existence of an acceleration signal for Parkinsonian rest tremor in the basal ganglia, in line with the “dimmer-switch” hypothesis.

## Introduction

Tremor is one of the cardinal symptoms of Parkinson's disease (PD). Parkinsonian tremor occurs primarily at rest, although a combination with postural and/or kinetic tremor is common (1). It affects about 75% of the PD patient population (2) and can range from mild to disabling. In case tremor cannot be sufficiently suppressed by pharmacological treatment, deep brain stimulation (DBS) of the subthalamic nucleus (STN) (3) or the ventral intermediate nucleus of the thalamus (4) is a commonly chosen treatment option.

Recently, it has been argued that continuous DBS, which is the standard today, may not be optimal for tremor control, since tremor is a dynamic symptom waxing and waning spontaneously. Adaptive systems capable of detecting tremor and stimulating on demand might be a more suitable alternative (5). Adaptive systems can use peripheral recordings for tremor detection (6, 7) or electrophysiological signals recorded by the DBS electrode. In fact, several studies have revealed patterns in brain activity associated with tremor, which might serve as a control signal in adaptive DBS systems. These include features of local field potential (LFP) oscillations in the STN, such as power and coherence at individual tremor frequency (8–10), beta power (13–30 Hz) (11, 12), low gamma power (31–45 Hz) (13, 14), and high frequency oscillations (>200 Hz) (15, 16).

While these signals are known to be associated with tremor, it is unclear whether they relate to tremor onset or to tremor maintenance specifically. These two processes are believed to be controlled by different subcortico-cortical circuits, according to a recently proposed model of tremor, called the “dimmer-switch” hypothesis (17). This hypothesis is based on a functional magnetic resonance imaging study showing that tremor amplitude is correlated with thalamic and cerebellar activity, whereas *changes* in tremor amplitude are correlated with pallidal activity (18). Thus, the authors proposed that the basal ganglia drive changes in the tremor state (the “switch”) whereas the cerebello-thalamo-cortical circuit maintains tremor and modulates its amplitude (the “dimmer”). According to this model, tremor onset should be associated with basal ganglia activity. This prediction can be validated by electrophysiology, which offers the appropriate temporal resolution to investigate tremor onset specifically.

The search for a specific marker of tremor onset is complicated by the fact that tremor appears and disappears on a very slow timescale. Intra- and perioperative, invasive recordings in patients, however, are usually time-constrained, such that most patients either exhibit continuous tremor during the measurement or no tremor at all. Here, we sought to overcome this problem by measuring many tremor onsets in a patient with the ability to interrupt tremor voluntarily. The measurements were obtained in several sessions within a period of 2 years using an implanted DBS system capable of LFP recording (Activa PC+S™, Medtronic, USA). The longitudinal design allowed us to (i) collect a larger number of tremor onsets and (ii) to monitor movement-related brain signals over a long period of time. For the latter part, we aimed at investigating the robustness of subcortico-cortical activity associated with a set of standard motor tasks and to relate the temporal development of this activity to the development of symptoms.

## Materials and Methods

### Patient

We report results from a single patient diagnosed with idiopathic Parkinson's disease of the tremor-dominant sub-type. The patient gave written informed consent and the study was approved by the local ethics committee (study no. 4326).

The patient (age range: 42–48 y) first noticed rest and action tremor in 2000, starting in the right leg and spreading to the other extremities, later accompanied by hypokinesia. Reduced dopamine transporter density was confirmed by single photon emission computed tomography. The patient showed a clear response to levodopa (MDS-UDPRS III Medication OFF/Medication ON: 43/28; highest score possible: 132) and no cognitive deficit (Mattis Dementia Rating Scale: 142/144, Montreal Cognitive Assessment: 30/30). Tremor, however, was only slightly improved by levodopa (pre-surgical daily dose: 600 mg) and did not respond to beta-blockers, primidone, clozapine, or topiramate. The patient was implanted with an Activa PC+S™ DBS system (Medtronic, USA) in 2015. DBS led to marked symptom suppression (MS-UDPRS III Medication OFF– DBS OFF/ Medication OFF—DBS ON: 50/25), including tremor, and initially allowed for a complete discontinuation of medication. Two years later, at the time of the last measurement, reemerging lower limb tremor required treatment with 50 mg of levodopa and 2 mg of rotiogotin in addition to DBS.

In Medication OFF/DBS OFF, the patient suffered from severe bilateral leg tremor when resting. Importantly for this study, the patient was able to interrupt leg tremor for several seconds by repositioning the feet.

### Experimental Paradigm

LFP and EEG signals were measured 1 week, 3 months, 6 months, 1 year, and 2 years after surgery. In order to limit battery usage, each LFP recording was constrained to 15 min. Anti-parkinsonian medication, if any, was withdrawn >12 h before the recordings started and DBS was turned off between 30 and 60 min before the recording started. Each session consisted of four motor tasks, referred to as REST, HOLD, MOVE, and TREMOR in the following. In the REST condition, the patient sat relaxed and quietly with eyes open for 2 min. In the HOLD condition, the patient elevated both forearms to 45° angle relative to the body's frontal plane and spread the fingers for 3 min. In the MOVE condition, the patient opened and closed the right fist (contralateral to LFP recording) in a self-paced fashion with a frequency of ~1 Hz for 3 min. In the TREMOR condition (7 min), the patient repeatedly interrupted bilateral rest tremor of the legs by repositioning the feet. Tremor reemerged spontaneously and was interrupted again after 20 s, upon verbal instruction of the experimenter. All sessions were recorded on video.

### Data Acquisition

The Activa PC+S™ system allows for chronic recordings from the DBS target site in addition to therapeutic stimulation (19). We used this system for longitudinal, bipolar LFP recordings from the left STN (contact 0 vs. contact 3). We used the first and the last electrode contact to record the largest area possible, thus reducing the risk of missing relevant subthalamic activity. The sampling rate was 794 Hz. In addition to the subthalamic LFPs, we recorded from eight scalp EEG electrodes (Cz, Fz, Pz, C3, Oz, FC2, P3, and P4) using a portable amplifier (Porti, TMSi, The Netherlands), and measured the vertical and the horizontal electrooculogram (EOG), the electrocardiogram (ECG), and the electromyogram (EMG) of the lower arms and of the lower legs on both body sides. These signals were sampled at 2,048 Hz. Before each recording, a transcutaneous, biphasic electric pulse with 5 mA amplitude and 2 ms duration was applied by an Osiris stimulator (Inomed, Germany). The pulse served as a common temporal marker visible in both the LFP and the EEG trace, and was used to align the signals in time during offline analysis (see below). It was delivered via two surface EMG electrodes, one attached to the neck and one above the subcutaneous cable connecting the DBS electrodes to the implanted pulse generator.

### Data Preprocessing

All analyses were carried out with Matlab (The Mathworks, USA) and the Matlab-based toolbox FieldTrip (20). The EEG was re-referenced to the average of all scalp EEG channels and band-stop filters were applied to remove 50 Hz line noise and its harmonics. EMG data were high-pass filtered at 10 Hz and rectified. EOG and ECG signals were demeaned. Subsequently, these signals were temporally aligned with the LFP signal, using the transcutaneous pulse as a common temporal marker. Next, bad channels and epochs with strong artifacts were removed. The EEG signal was decomposed using FASTICA (21), components reflecting heart beat and eye movements were deselected manually and the signal was back-transformed.

### Spectral Analysis

For the computation of epoch-average spectra, data were cut into segments of 1 s duration and 50% overlap. Segments were convolved with a Hanning taper and subjected to Fourier transformation. The Fourier coefficients were used to compute power and coherence spectra. For the computation of time-frequency representations (TFRs), Fourier coefficients were computed for each time-frequency bin using Morlet wavelets with a width of 7. TFRs were baseline-corrected (−0.5 to −0.15 s relative to tremor onset).

Although we analyzed the data up to the highest frequency possible (397 Hz), we only show spectra up to 45 Hz in this report because tremor-specific effects occurred in this range. Furthermore, we opted to represent the cortical signal by the Cz-electrode, which showed the strongest coherence with the LFP.

### Epoch Selection

#### Tremor Onset

**Figure 1:**
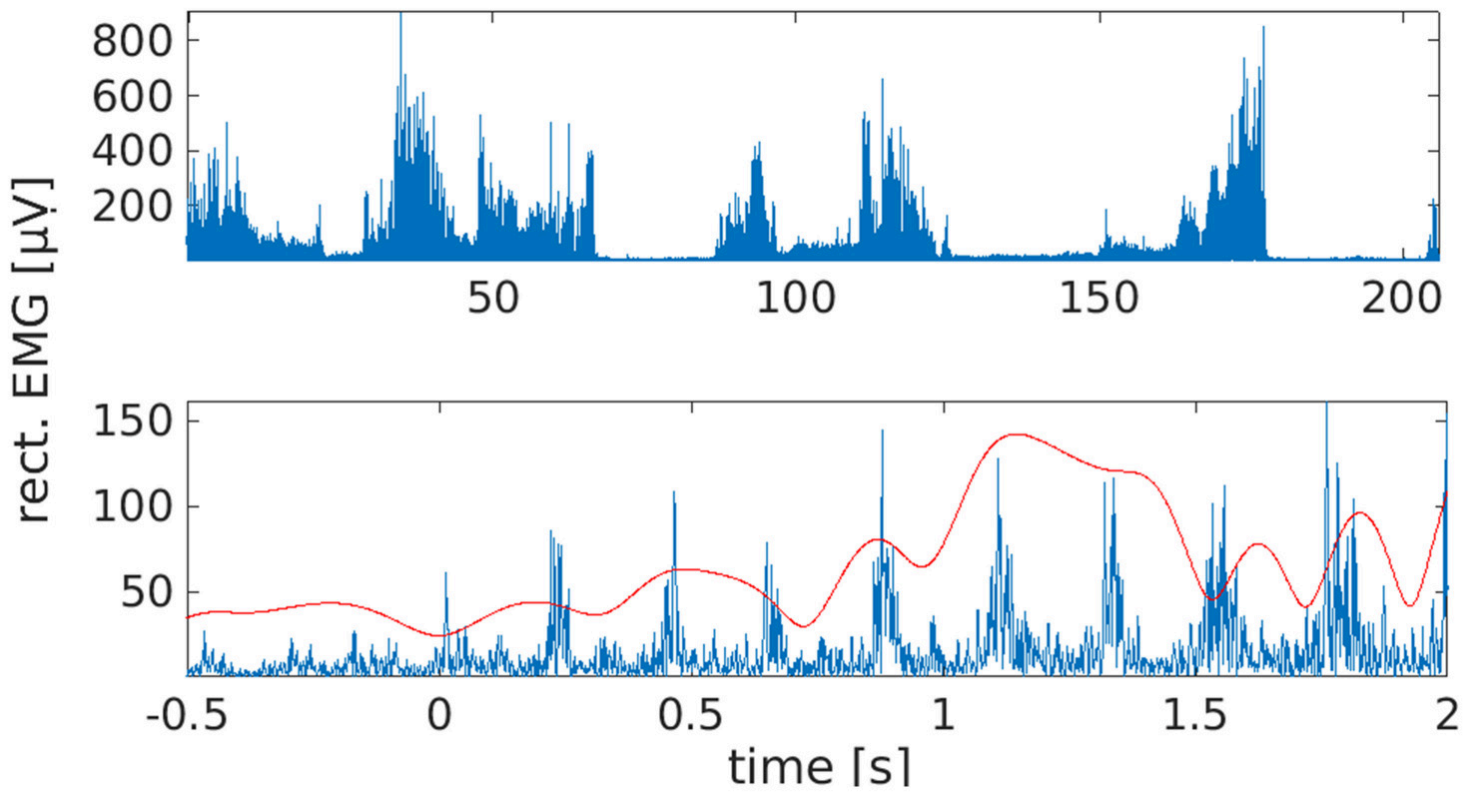
Waxing and waning of leg tremor. **(Top)** Rectified EMG trace from right lower leg. Note the presence of transient tremor arrests/reductions. These were induced by the patient by repositioning the feet. **(Bottom)** Example of a tremor onset episode. Time 0 marks the time of tremor onset, as estimated by inspection of the right leg EMG trace (blue) and EMG power between 3 and 8 Hz (red).

Although we recorded about 100 attempted tremor arrests in total, we included only those 38 trials in the analyses with a clearly identifiable onset. The latter was not always observable because voluntary tremor arrest was not always successful. Tremor onsets were identified by first screening leg EMG traces and right leg EMG power between 3 and 8 Hz, as obtained by Hilbert transformation, and by marking all potential leg tremor onsets. The band limits for EMG power were chosen to accommodate both the individual tremor frequency (3.5 Hz) and its first harmonic (7 Hz). Following the labeling of candidate onsets, we checked whether leg tremor was suppressed and the muscle was relaxed for at least 500 ms. If this was the case, we searched the right leg EMG power trace for the last local minimum before tremor reemergence and defined this sample as tremor onset (Figure 1). Onset times were confirmed by visual inspection of the time-domain EMG signal. For spectral and statistical analysis, tremor onsets from all sessions were pooled to maximize statistical power.

Information on onset detection for voluntary movements (feet repositioning and fist-clenching) is provided in the Supplementary Material.

#### Tremor Episodes and Tremor-Free Episodes

To investigate subthalamic and cortical signals during tremor maintenance, we inspected the EMG recording of the right lower leg and marked tremor episodes and tremor-free episodes. The other EMG signals were disregarded, but the left and the right leg were equally affected by tremor. Hence, the terms “tremor episode” and “tremor-free episode” relate to leg tremor, but not to tremor in other body parts. Again, episodes from all sessions were pooled to maximize statistical power. Note that the lower leg musculature was relaxed during tremor-free episodes, i.e., these episodes do not reflect tremor suppression through muscle contraction.

### Statistics

Spectra were compared using FieldTrip's cluster-based permutation approach (22), which corrects for testing at multiple frequencies or time-frequency bins. In each comparison, the number of epochs/trials was equalized across experimental conditions by deleting the last samples from the longer condition.

#### REST vs. HOLD vs. MOVE

For each session, logarithmic power and coherence in the conditions REST, HOLD, and MOVE were compared pairwise using a *t*- (power) and a *z*-statistic (coherence) (23) for independent samples, respectively. The resulting *p*-values, already corrected for testing at multiple frequencies, were further corrected for comparing at multiple dates using False Discovery Rate.

#### Pre-onset vs. Post-onset

TFRs of logarithmic LFP and EEG power were compared between the pre-onset baseline (−0.65 to −0.15 relative to tremor onset) and the post-onset phase using the “activation vs. baseline *t*-statistic” (22). For this method to work, the baseline and the post-onset epoch need to have equal length. The length of the baseline epoch was dictated by the shortest tremor-free episode (500 ms). Hence, we set the post-onset epoch to 500 ms and shifted this window by 500 ms four times, thus probing a 2 s period following tremor onset in four separate tests. We did not test coherence this way because a corresponding statistic for coherence is not available. We do, however, provide time-resolved plots of coherence to give an impression of phase difference consistency across tremor onsets for each time-frequency bin.

#### Tremor vs. Rest

Tremor and tremor-free episodes were compared in the same way as REST, HOLD, and MOVE. We did not apply False Discovery Rate, however, since data were pooled over sessions, i.e., we did not perform separate comparisons at each date.

## Results

### Longitudinal Measurements of Subcortico-Cortical Activity in Different Motor Tasks

**Figure 2:**
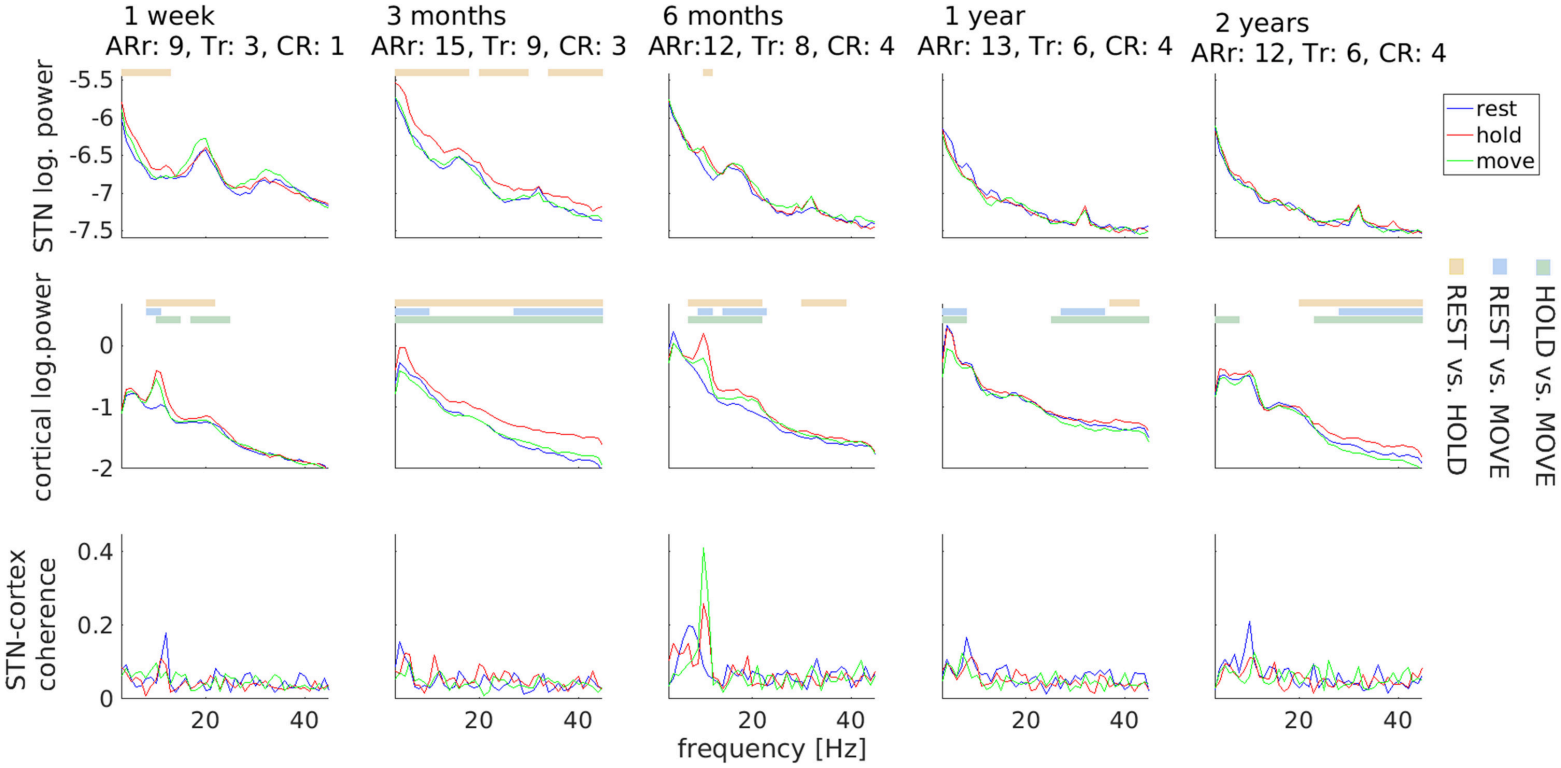
Temporal stability of subthalamic and cortical oscillations. Motor tasks are color-coded. Horizontal lines mark significant differences and comparisons are coded by another color scheme (see legends on the right). Values underneath the row titles indicate body side-specific scores for akinesia/rigidity (ARr; UPDRS III items 3.3–3.8) and for tremor (Tr: UPDRS III items 3.17) for the right body side, i.e., contralateral to local field potential recordings, and for the constancy of rest tremor (CR: UPDRS III item 3.18). Clinical scores obtained 3 months before surgery: ARr: 10, Tr: 8, CR: 3.

Clinically, DBS surgery resulted in a marked, transient symptom reduction (stun effect), particularly of tremor (Figure 2). Three months before surgery, the UPDRS III sum-score for Medication OFF/DBS OFF was 43. It decreased to 29 one week after surgery, and increased again to 57 three months after surgery. Thereafter, it remained on a comparable level (six months: 52, one year: 50, two years: 50). Interestingly, STN beta power underwent a different temporal development. It was strongest 1 week after surgery, when tremor was suppressed due to the stun-effect, but diminished over time.

With respect to electrophysiology, the HOLD condition was repeatedly associated with more power than REST and MOVE in a variable frequency range. This difference was most prominent for the alpha peaks observed in the first-week and sixth-month recordings. The most robust electrophysiological feature was a peak in STN-cortex coherence around 10 Hz, which was visible in all sessions.

Note that differences between motor tasks were only observable up to 6 months after surgery in the LFP signal, indicating a possible decrease of the signal-to-noise-ratio over time. We evaluated the available data on contact impedance and observed an increase occurring earlier than 6 months. The impedance of contact L03 was 1726 Ω at the day of stimulator implantation, 4,261 Ω 3 months after surgery, and 3,153 Ω 2 years after surgery. In other words, the loss of LFP beta peaks after 1 year was not accompanied by a corresponding increase in impedance.

### Tremor Onset

**Figure 3:**
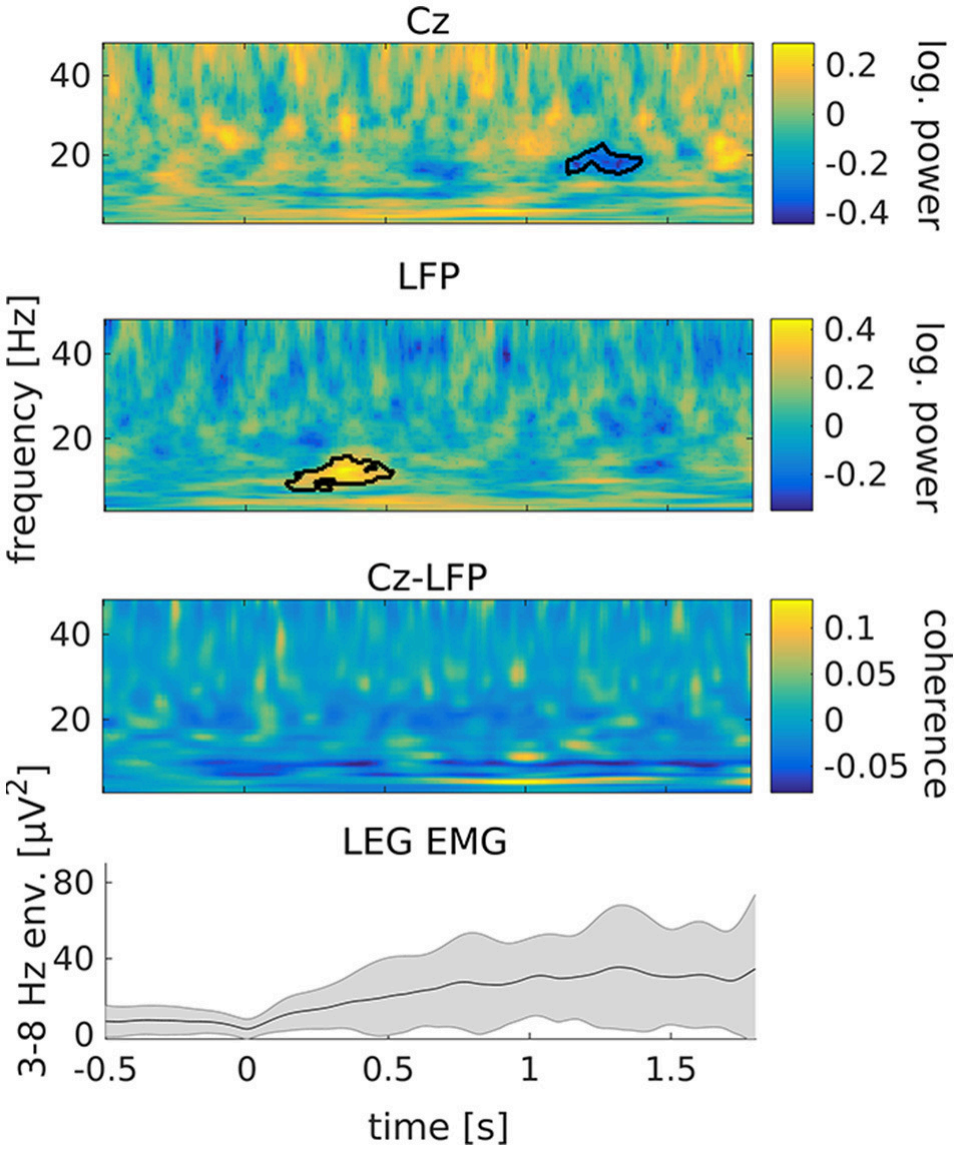
Tremor onset is associated with a transient increase of subthalamic power at alpha/low beta frequencies. From top to bottom: cortical power, STN power, STN-cortex coherence and leg EMG power between 3 and 8 Hz. Time 0 marks tremor onset. Colors indicate the difference to baseline (−0.65 to −0.15 s relative to tremor onset). The black outline marks significant differences. The gray shading in the lowermost plot illustrates the standard deviation across tremor epochs.

Following tremor onset, we observed a significant increase of STN power between 8 and 15 Hz compared to pre-onset baseline (Figure 3; *p* = 0.009). This increase occurred between 150 to 520 ms after tremor onset. At this stage, tremor had not evolved to full amplitude in most of the epochs yet, as indicated by the dynamics of leg EMG power (Figure 3, lowermost plot). Cortical beta power between 16 and 20 Hz decreased compared to pre-onset baseline at 1.15–1.4 s relative to tremor onset (*p* = 0.019). Furthermore, we observed an increase of STN-cortex coherence at 5 Hz, following the occurrence of the transient alpha/low-beta power increase in the STN.

We tested the tremor-specificity of the STN power increase between 8 and 15 Hz by examining STN power changes around two types of voluntary movement: feet repositioning and repetitive fist-clenching. Feet repositioning was associated with a post-movement increase of beta-band power around 20 Hz, possibly related to tremor arrest (Figure S1 of the Supplementary Material). A power increase between 8 and 15 Hz was not observed. Repetitive fist-clenching showed a pattern of power changes that was in part reminiscent of tremor onset, including signs of an alpha/low-beta power increase around 300 ms after movement onset (Figure S3). This change, however, was not significant (*p* = 0.33) and much weaker than for tremor onset.

### Tremor Maintenance

**Figure 4:**
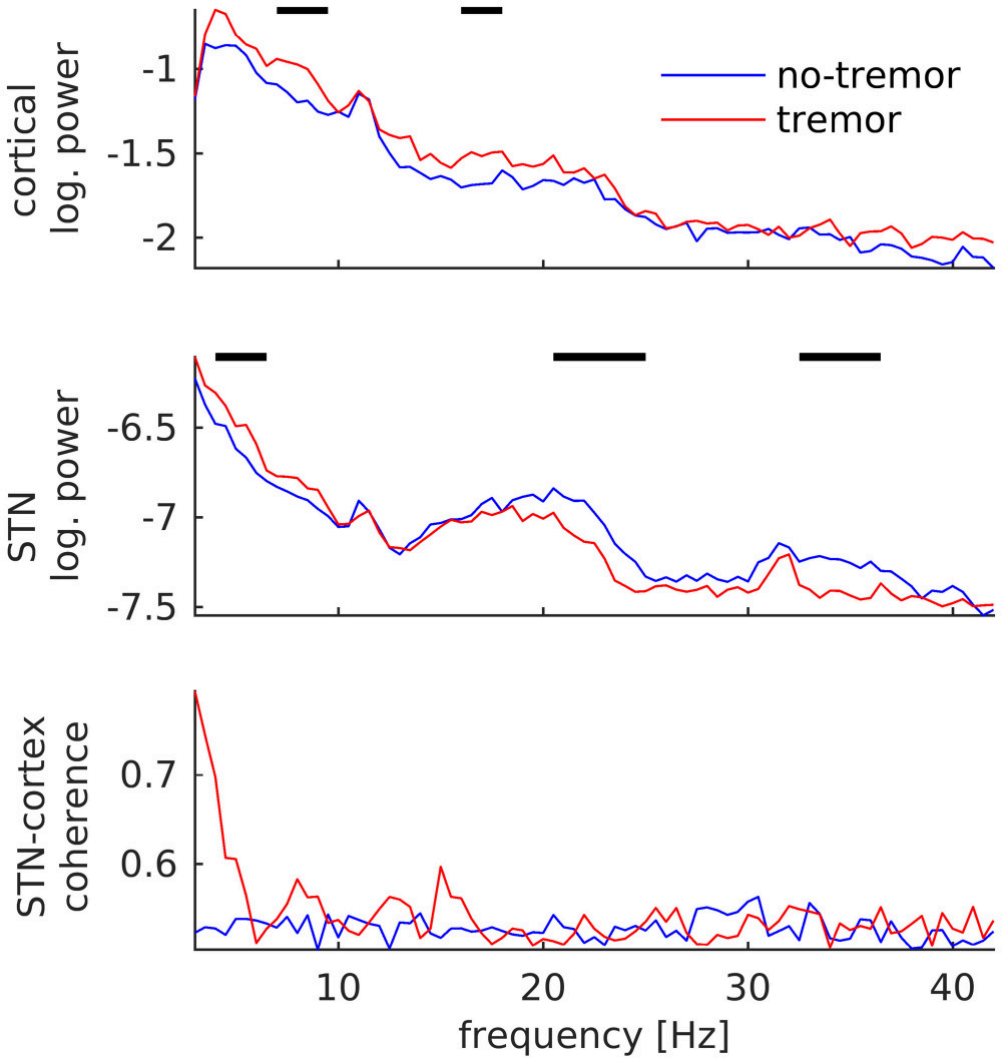
Changes of subthalamic and cortical signals during tremor maintenance. Cortical **(Top)** and subthalamic **(Middle)** power averaged over all tremor (red) and no-tremor segments (blue). **(Bottom)** STN-cortex coherence. Vertical bars mark significant differences.

In contrast to tremor onset, tremor maintenance was associated with a decrease, not an increase, in subthalamic beta oscillations. This decrease was observed for beta peaks in the low (20–25 Hz; *p* < 0.001) and the high beta/low gamma band (32–36 Hz; *p* < 0.001), as illustrated in Figure 4. In addition, we detected an increase of STN power between 4 and 7 Hz (*p* = 0.003), and increases of cortical power between 7 and 10 Hz (*p* < 0.001) and 16 and 18 Hz (*p* = 0.004). STN-cortex coherence did not change significantly, but exhibited several peaks below 20 Hz in the tremor condition only.

## Discussion

### A New Maker of Tremor Onset

We have revealed an electrophysiological, subthalamic marker of tremor onset, suggesting that the basal ganglia mediate the emergence of tremulous movements, as proposed by the “dimmer-switch” hypothesis (17). Interestingly, the “dimmer-switch” hypothesis does not specify whether the STN is part of the “dimmer” (cerebello-thalamo-cortical loop) or the “switch” (striato-pallidal loop). This is because the STN is part of the basal ganglia but also receives projections from and sends projections to the cerebellum (24). Indeed, the current results implicate a dual role for the STN. Subthalamic beta power was found to be modulated both at tremor onset and during tremor maintenance, albeit in opposite directions. These observations suggest that distinct processes are at work during tremor onset and tremor maintenance, and the STN is involved in both of them.

In addition to revealing a new marker of tremor onset, the current results confirm beta power suppression and increases at individual tremor frequency and/or its harmonics as basic markers of tremor maintenance (10–12, 25). Note that the current results differ partly from our own previous work on Parkinsonian rest tremor (10), which investigated consistent patterns of power and coherence in 11 PD patients. This work had revealed an increase of STN power at individual tremor frequency occurring about 1 s after tremor onset and lasting for the entire epoch under investigation (9 s), but it had not detected the 8–15 Hz increase reported here. In light of the current findings, we interpret the late and sustained increase of subthalamic power at individual tremor frequency as a correlate of tremor maintenance, which is reflected in the 4–7 Hz increase in the STN power spectrum in this study (Figure 4, upper row). The transient 8–15 Hz increase might have been missed in our previous study due the higher variability introduced by sampling from different patients. The current study, in turn, did not find a significant increase of STN-cortex coherence during tremor, but the pattern observed here matches our previous results on a qualitative level. Finally, we observed a tremor-associated increase in cortical oscillations in the beta band and below, although previous studies reported a beta power decrease (12). This discrepancy might be due to tremor-related EEG artifacts in the data at hand, which can arise during severe tremor (several seconds after tremor onset) and are difficult to remove completely.

In addition to beta oscillations and oscillations at individual tremor frequency, high-frequency oscillations (HFOs; >200 Hz) were found to reliably reflect the tremor state of PD patients (15, 16, 25). Unfortunately, we did not find any HFO peaks in the data at hand, most likely because the amplitude of HFOs is too low for detection by the PC+S™ system.

### Insights From Longitudinal Recordings

To our knowledge, this is the first longitudinal, invasive recording of tremor-related patterns in power and coherence in the STN of PD patients. Previous studies on PD have utilized the Activa PC+S™ system for recording changes of STN power associated with voluntary or passive movements in patients (26–28) and in non-human primates (29, 30), and for investigating the effects of DBS on STN power (31, 32). In addition, Swann and colleagues have revealed a relationship between dyskinesia and gamma oscillations in the STN and motor cortex (33). Finally, a recent report made use of PC+S™ data to fit a computational model of LFP rhythm generation (34).

The longest time range investigated in any of the previous patient studies was 12 months. Here, we performed measurements up to 24 months after surgery and found a 10 Hz peak in STN-coherence in all sessions, indicating that relevant, physiological signals remain measurable within this time frame. The fact that differences in STN power between motor tasks were only observed up to 6 months, however, suggests that the signal-to-noise ratio may have decreased over time. A similar decrease has been described in a previous study on cortical LFPs in non-human primates, using sub-dural electrodes (30). In this case, the deterioration of signal quality was associated with a dramatic increase of contact impedance and was found to be caused by regrowth of the dura. In our case, the loss of STN beta peaks observed at 1 year after surgery and thereafter was not accompanied by a corresponding increase in contact impedance, suggesting a minor role of tissue reorganization at the electrode-tissue interface.

A noteworthy finding of the longitudinal analysis is that a prominent beta power peak was visible in the first week after surgery but vanished over time. During the first week, the patient experienced a strong, transient reduction of motor symptoms caused by electrode insertion (stun effect). The stun effect has previously been used to explain the lack of beta peaks in some PD patients (31, 35). This study suggests that the stun effect can also lead to an increase rather than a disappearance of beta peaks, associated with tremor suppression. In agreement with this idea, the constancy of rest item of the UPDRS and STN beta power underwent antagonistic developments (Figure 2). Alternatively, the decline of power at beta frequencies might be a general marker of disease progression (36) or might be caused by a decrease in the signal-to-noise ratio not accompanied by increased impedance (see above). It could also be an after-effect of chronic DBS (32).

### Clinical Relevance

Electrophysiological markers of PD symptoms are clinically relevant because they might be used to control adaptive DBS systems applying stimulation based on the current clinical state rather than continuously. The Activa PC+S™ system, for example, can be incorporated into such systems (37). Adaptive stimulation can either be controlled by brain signals measured by the DBS electrodes or by external signals, such as accelerometers (6, 7). Studies using prototypes of adaptive, systems conditioned on brain activity suggest increased clinical benefit (38–40) and reduced occurrence of side-effects (5, 41) as compared to conventional DBS. The robustness of the relevant electrophysiological symptom markers is a prerequisite for the application of adaptive DBS systems (42), and longitudinal measurements, as performed in the current study, can provide information about their long-term stability.

This study adds a short, transient increase of subthalamic alpha/low-beta power to the list of potential markers of tremor. The short latency (~150 ms) of this signal relative to the first noticeable sign of tremor in the EMG might enable adaptive systems to prevent tremor manifestation at an early stage, possibly before the patient is aware of tremor and certainly before tremor is noticed by others or interferes with voluntary movements. Of course, noticing the brief alpha/low-beta power increase would require a very sensitive detector. Detector specificity, on the other hand, would depend on whether STN alpha/low-beta power undergoes comparable changes in situations other than tremor onset. Although the current study cannot provide a final answer to this question, we have not seen increases of alpha/low-beta power at comparable strength in voluntary foot or hand movements, suggesting that tremor-specificity is achievable.

### Limitations

With only a single patient, the current study is not representative of the PD patient population. In particular, the patient under study suffered from severe leg tremor in addition to upper limb tremor, which is not the most common symptomatology. Future experiments need to investigate to what degree the results generalize to other muscles, to other patients, and to other types of tremor.

Furthermore, although we have been very careful in the definition of tremor onset, inspection of EMG traces cannot provide unequivocal onset times. Hence, the onset times and the latencies reported here need to be understood as estimates rather than precise measurements.

## Ethics Statement

The patient gave written informed consent and the study was approved by the local ethics committee (study no. 4326).

## Author Contributions

AS and LW designed the experiment. LS and CH collected clinical data. OA, LS, JH, and MB recorded the electrophysiological data. JH analyzed the data and wrote the first draft of the manuscript. AS, MB, LW, OA, and CH reviewed and edited the manuscript.

### Conflict of Interest Statement

AS and LW have received consultant/speaker fees from Boston Scientific, Medtronic, Inomed, and/or Abbott. The remaining authors declare that the research was conducted in the absence of any commercial or financial relationships that could be construed as a potential conflict of interest.
