# Supplementary material for: Longitudinal Recordings Reveal Transient Increase of Alpha/Low-Beta Power in the Subthalamic Nucleus Associated With the Onset of Parkinsonian Rest Tremor
Source: Front Neurol. 2019 Mar 7;10:145. doi: 10.3389/fneur.2019.00145 (PMC6416159; doi:10.3389/fneur.2019.00145)
Supplement: Supplementary file 1 [file Data_Sheet_1.pdf]

Supplementary Material

Longitudinal recordings reveal transient increase of alpha/low-beta power in the subthalamic nucleus associated with the onset of Parkinsonian rest tremor

In the main article, we present an STN power increase between 8 and 15 Hz occurring shortly after tremor onset. Here, we test the tremor-specificity of this signal by comparing it to STN power changes occurring around two types of voluntary movement: feet repositioning and repetitive fist-clenching.

Feet repositioning

Feet repositioning onset was determined by visual inspection of the right leg EMG trace. The event was difficult to identify due its embedding in large-amplitude leg tremor, and we selected only clearly discernable events. Furthermore, we restricted the analysis to voluntary foot movements leading to tremor arrest, to avoid a mix of tremor-related and movement-related signals in the post-onset period. An example of such an event is provided in **Fig. S1A**. The selection procedure resulted in 19 trials in total. The trial-average time-frequency representation of STN power around feet repositioning is depicted in **Fig. S1B**.

The most prominent feature observed was a post-movement power increase around 20 Hz. Given that STN beta power is known to be suppressed by tremor (1,2), this feature is likely related to tremor arrest. Alternatively, it might be a post-movement beta rebound following foot movement (but note that beta rebounds are typically overshooting the baseline transiently (3,4)). Importantly, we did not find an increase of power between 8 and 15 Hz, as observed during tremor onset. This finding suggests that the alpha/low-beta power increase reported in the main paper is related to tremor onset rather than to voluntary foot movement.

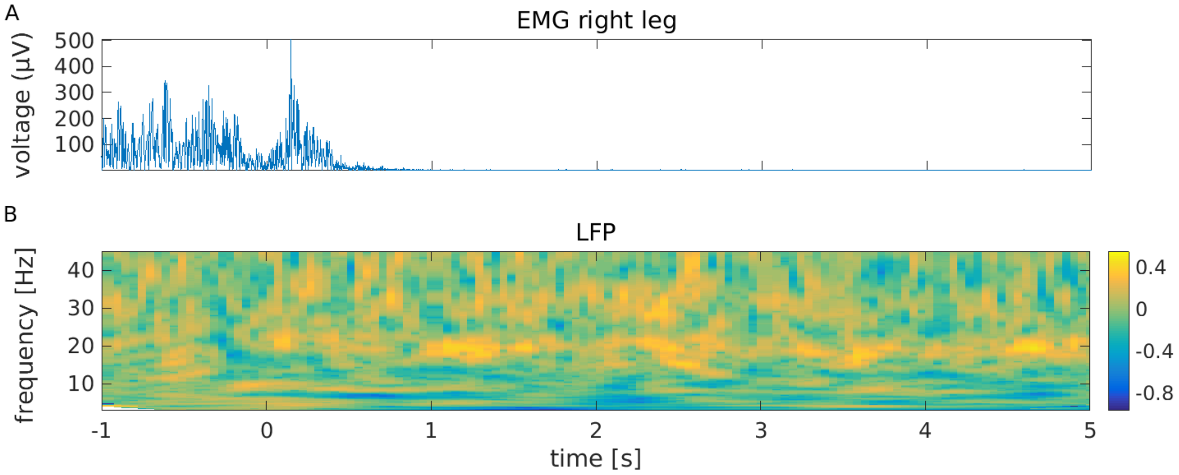

Fig S1: ***Changes of subthalamic power around voluntary foot movements.*** Time 0 marks foot movement onset. **A)** Example of the right leg EMG trace in successful tremor arrest. **B)** Time-frequency representation of logarithmic, trial-average power. Difference to baseline (-1 to -0.1 s) is color-coded.

Repetitive fist-clenching

The patient performed 3 min of self-paced, repetitive fist-clenching with the hand contralateral to STN recordings in each of the 5 sessions. Here, we focused on the one-week session as the patient exhibited little to no kinetic tremor during fist-clenching solely in this recording. Movement onset was detected automatically. First, we computed the absolute Hilbert transform of the EMG of the right flexor digitorum longus muscle, low-pass filtered at 3 Hz. We then applied a Savitzky-Golay filter (frame length: 398 samples, order: 3) to smooth this signal and to compute its first derivative. Next, we used Matlab's *findpeaks* function to identify local maxima in the derivative, i.e. moments of maximal velocity. These corresponded well to movement onset estimates obtained through visual screening (**Fig. S2**) and were thus used for definition of hand movement onset. The selection procedure resulted in 268 trials in total.

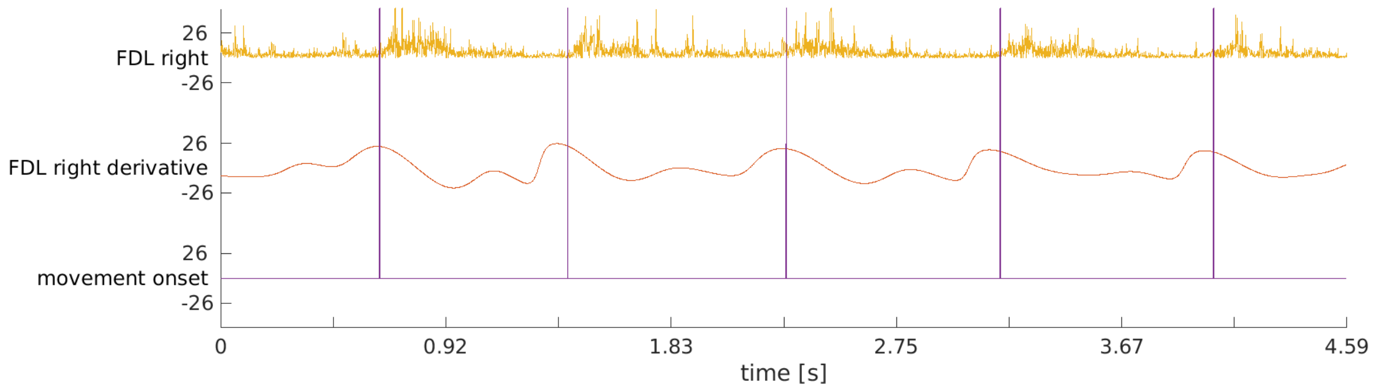

Fig. S2: ***Automatic detection of fist-clenching onset***. Top: rectified EMG trace of the right flexor digitorum longus (FDL) muscle during repetitive fist-clenching. Middle: Derivative of the smoothed FDL EMG power envelope. Bottom: Automatically detected movement onset times. Signals were re-scaled for illustration; units are arbitrary.

Fig. S3 depicts the trial-average STN power changes around hand movement onset in comparison to leg tremor onset. Qualitatively, there were similarities between tremor onset and voluntary hand movement, particularly below 20 Hz. Alpha/low-beta power increased around 300 ms in both conditions, but the increase was much more pronounced for tremor onset than for voluntary movement (note the different scales). Comparing the 500ms epochs immediately preceding and following movement onset, respectively, yielded a significant difference for tremor onset only (tremor onset: p = 0.009, fist-clenching: p = 0.33). These findings suggest that the high amplitude of the alpha/low-beta power increase might be a sign of tremor, rather than the increase itself.

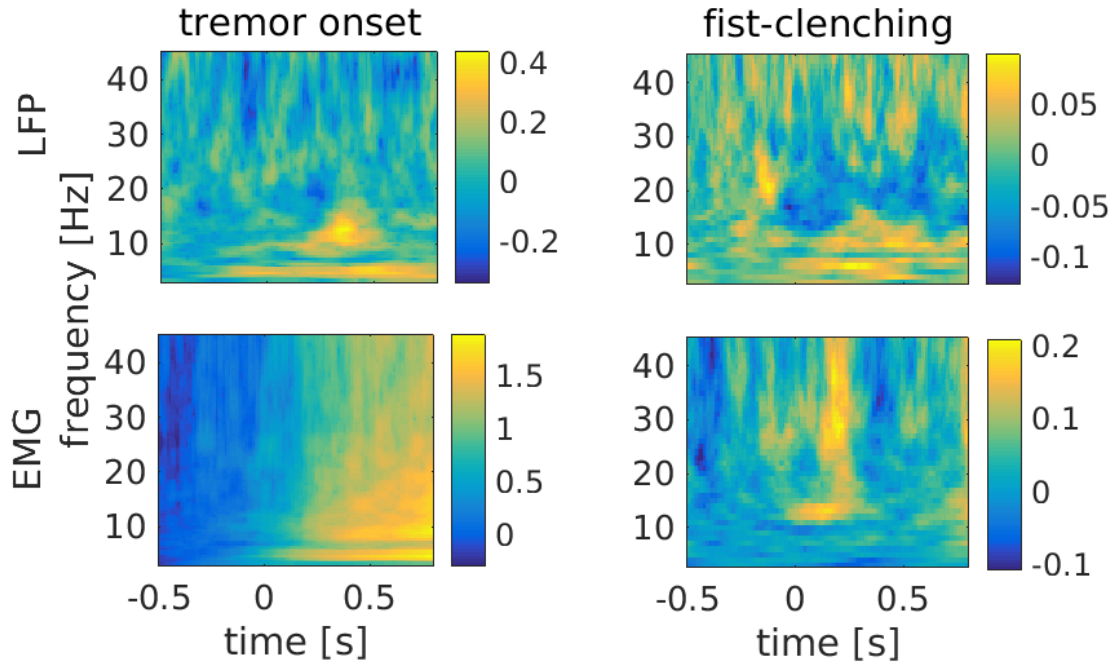

Fig. S3: ***A comparison of STN power changes around leg tremor onset (left) and voluntary hand movement (right).*** Top: Local field potential power recorded from the subthalamic nucleus. Bottom: EMG power of the right leg (left) and of the right forearm (right). The difference in logarithmic power to baseline (-0.5 to 0.7 s) is color-coded. Time 0 marks movement onset.

References:

1. Wang SY, Aziz TZ, Stein JF, Liu X. Time-frequency analysis of transient neuromuscular events: Dynamic changes in activity of the subthalamic nucleus and forearm muscles related to the intermittent resting tremor. *J Neurosci Methods* (2005) **145**:151–158. doi:10.1016/j.jneumeth.2004.12.009

2. Qasim SE, de Hemptinne C, Swann N, Miocinovic S, Ostrem JL, Starr PA. Electrocorticography reveals beta desynchronization in the basal ganglia-cortical loop during rest tremor in Parkinson's disease. *Neurobiol Dis* (2016) **86**:177–86. doi:10.1016/j.nbd.2015.11.023

3. Storzer L, Butz M, Hirschmann J, Abbasi O, Gratkowski M, Saupe D, Vesper J, Dalal SS, Schnitzler A. Bicycling suppresses abnormal beta synchrony in the Parkinsonian basal ganglia. *Ann Neurol* (2017) **82**:592–601. doi:10.1002/ana.25047

4. Litvak V, Eusebio A, Jha A, Oostenveld R, Barnes G, Foltynie T, Limousin P, Zrinzo L, Hariz MI, Friston K, et al. Movement-related changes in local and long-range synchronization in Parkinson's disease revealed by simultaneous magnetoencephalography and intracranial recordings. *J Neurosci* (2012) **32**:10541–53. doi:10.1523/JNEUROSCI.0767-12.2012
